# Steroids Decrease Prevalence of Positive Tuberculin Skin Test in Rheumatoid Arthritis: Implications on Anti-TNF Therapies

**DOI:** 10.1155/2014/430134

**Published:** 2014-02-24

**Authors:** Shweta Agarwal, Siddharth Kumar Das, Girdhar G. Agarwal, Ragini Srivastava

**Affiliations:** ^1^Department of Rheumatology, King George Medical University, Lucknow, Uttar Pradesh 226018, India; ^2^Department of Statistics, Lucknow University, Lucknow 226007, India

## Abstract

Tuberculin skin test has been used as an indicator of latent tuberculosis in patients with Rheumatoid Arthritis (RA) before administration of biologicals. Effect of Disease modifying antirheumatic drugs (DMARDs) and steroids on the result of tuberculin skin test (TST) may have important implications in interpretation of results of this test. *Objectives*. To find the prevalence of positive TST in rheumatoid patients and the effect of standard treatment on the results of TST. *Method*. In this cross-sectional study two hundred and fifty patients of RA above 18 years of age, classified using 1987 ACR criteria for RA, were enrolled from rheumatology outdoor. Demographics, disease activity, disease duration, and therapy were recorded. All patients underwent TST. *Results*. Fifty-one (20.4%) patients were found to be tuberculin positive. Tuberculin positivity was not affected by MTX intake but it was significantly low in patients with recent steroid intake as compared to patients who had not taken steroids in last 3 months (3% versus 25%, *P* = 0.002). *Conclusion*. Prevalence of tuberculin positivity in patients with RA was found to be low. Results were not affected by methotrexate; however tuberculin skin test results in patients with recent use of steroids are likely to be negative.

## 1. Introduction

The association between rheumatoid arthritis (RA) and tuberculosis (TB) dates back to more than nine decades ago, when several clinicians in Germany and Forrestier in France related the chronic inflammation of tuberculosis to that of rheumatoid arthritis and thus used gold salts for the treatment of RA in the 1920s [1, 2] based on the fact that aurothiosulfate sodium was effective in the treatment of pulmonary tuberculosis [3]. Bahr et al. even emphasized the role of cross-reactive mycobacterial antigens in etiopathogenesis of RA [4].

With time as further researches negated the etiological role of mycobacteria in RA, the relationship between the two took a turn so that the occurrence of two diseases together was not considered to be dependent on each other. With the advent of steroids and DMARDs in the treatment of RA, researchers started debating the increased incidence of tuberculosis in RA due to immunosuppression. A large observational cohort study from Japan reported a 3.2-fold increased risk of tuberculosis in patients with RA treated with standard therapy [5]. Similar results have been reported from Spain and Sweden even before the anti-TNF era whereas studies from US reveal no difference in incidence of tuberculosis between the general population and those suffering from RA on standard therapy [6–8]. Further with the introduction of biologicals for the treatment of RA, the issue of association between the two again gained acceleration. Various studies suggest 5- to 10-fold increased risk of reactivation of latent TB with the use of anti-TNF antibodies [9–12].

Tuberculin skin test (TST) has been used as indicator of latent tuberculosis (LTBI) in patients with RA before administration of biologicals. Though there are studies regarding effect of RA therapy on incidence of tuberculosis, effect of DMARDs, which are used as standard therapy in patients of RA, on the result of tuberculin test is not known. This effect may have important implications on interpretation of results of tuberculin test prior to biological therapy. This study was thus undertaken with the aim of finding the prevalence of positive TST and effect of standard treatment (DMARDs and steroids) on the results of TST in patients with rheumatoid arthritis.

## 2. Material and Method

Patients, for this observational, cross-sectional, and prospective study were recruited from outpatient department of Department of Rheumatology at King George's Medical University, Uttar Pradesh, Lucknow, India. Prevalence of tuberculosis in India has been reported as 256 per one hundred thousand population by Central TB Division, India, in its annual status report 2013 [13]. Prevalence of latent tuberculosis in India has been reported to be around 38% in various studies [14, 15]. As far as the BCG penetration is considered, universal BCG vaccination started in India in the late 50s and currently all children are vaccinated with BCG. Study was approved by the Institutional Ethics Committee of KG Medical University, Lucknow. Informed consent was obtained from all the patients before any study related procedure. Sample size was calculated hypothesizing decreased prevalence of tuberculin sensitivity in patients with RA (25%) as compared to that in general population (38%) [14, 15]. Patients with rheumatoid arthritis (RA) both males and females above 18 years of age, classified using 1987 ACR criteria for RA [16], were enrolled over a period of 20 months (August 2011 to March 2013). All consecutive patients with RA, not already enrolled in our study, attending the Rheumatology clinic on each clinic day of our unit, giving consent and fulfilling the inclusion and exclusion criteria were enrolled regardless of the intake of therapy. Patients with any other connective tissue disorder or arthritides other than RA, any immunosuppressive condition, and hematological malignancies, those on biologicals, pregnant females, and patients with active tuberculosis or past history of tuberculosis were excluded.

Demographics, disease activity (DAS 28 - ESR score), disease duration, and therapy were recorded. DAS 28 Score < 2.6 was defined as remission, <3.2 as low disease activity, <5.1 as moderate disease activity, and ≥5.1 as high disease activity. Patients exposed to tobacco in form of tobacco chewing or active or passive smoking were classified as smokers while those not exposed to tobacco were classified as nonsmokers. All patients were examined clinically, subjected to digital X-ray chest PA view, and investigated as required to exclude active tuberculosis. Every patient underwent tuberculin skin test. Purified protein derivative (PPD) 1 TU (0.1 mL) was injected on the flexor aspect of forearm. Preparation used was “PPD-RT-23 with Tween 80”. Results were read after 72 hours. Tuberculin test was considered positive if induration exceeded 10 mm. It was considered negative if it is less than 5 mm and doubtful if between 5 and 10 mm.

Patients were divided into five groups on the basis of methotrexate (MTX) intake within last three months (control MTX: patients who had not taken MTX, A-patients on MTX ≤ 7.5 mg/week, B-patients on MTX >7.5–15 mg/week, C-patients on MTX >15–22.5 mg/week, and D-patients on MTX >22.5 mg/week). According to the steroid intake patients were divided into three groups (control S-patients without any steroid intake within last three months, I-patients with recent steroid intake defined as intake of any dose of steroid in any form (oral, intramuscular, or intra-articular) within 1 week prior to tuberculin test, and II-patients with history of steroid intake within last three months but not in last one week).

Statistical analysis was performed using SPSS 16.0 software. Chi-square test was used to test the hypothesis about equality of proportion between the groups. Fisher's exact test was applied where expected value of a cell was less than 5. For comparing means of continuous variables, Student's *t*-test was used.

## 3. Results

A total number of 300 patients, found eligible for the study, were invited to participate in the study from August 2011 to March 2013. Only 256 patients consented out of which six patients did not turn up for TST reading after 72 hours and were thus excluded making the total number of participants in the study 250. Primarily the patients were middle aged (47.2 ± 10.9 years), females (85.2%), nonsmokers (77.2%) with a mean duration of RA of 80.8 ± 63.7 months and moderate disease activity (43.6%) at study entry. 69.2% of the patients were found to be positive for rheumatoid factor. None of the patients had undergone a TST within last two years. Demographic profile of different groups of patients based on methotrexate dose is given in Table 1. Demographic profile of the patients with recent steroids was similar to that of the patients not taking steroids in terms of age (*t* = 0.44, *P* = 0.66), smoking status (Chi-square = 0.69, *P* = 0.41), rheumatoid factor positivity (Chi-square = 0.09, *P* = 0.76), disease duration (*t* = 1.73, *P* = 0.09), and disease severity (*t* = −1.33, *P* = 0.18) (Table 2).

Fifty-one (20.4%) of studied patients were tuberculin positive and 187 (74.8%) were negative while 12 (4.8%) had doubtful results. Though tuberculin positivity was not affected by MTX intake in patients (Table 3), it was significantly lower in patients with recent steroid intake (Group I) (*P* = 0.002), as compared to patients without any steroid intake within 3 months (control S). There was no significant difference between control S and group II (Table 4).

## 4. Discussion

According to a WHO report one-third of the world's population is believed to harbour a latent tuberculosis infection [17]. In Antalya district, Turkey, among the BCG unvaccinated group TST positivity was found to be none in 5–7-year, 2% in 14–25-year, and 7% in > or = 60-year age groups [18]. In a study conducted in 2007, the prevalence of positive tuberculin skin test was found to be 22% in Sweden [19]. A prospective study of serial tuberculin skin testing performed on 642 patients from the chronic care wards of a Veterans Administration Hospital revealed a positive test in 23.6% [20]. In a study in Taiwan, 34.2% of the study population tested positive for the TST [21]. From India, Seal et al. reported the prevalence of tubercular infection to be 38.9% in 1954 [14] and Narian et al. reported the similar figures in 1963 (38.3%) [15]. Prevalence rate of tuberculous infection in a rural population of South India was found to be 30% in 1968 (among females 25% and males 35%) [22]. In our study, 20.4% of patients with rheumatoid arthritis were found to be positive for TST. The study was done at a single centre in North India and may need to be replicated on multicentric study. Comparing the results from our study with the available population data, we can infer that presence of rheumatoid arthritis influences the results of tuberculin test in our population. In a study from Italy also, prevalence of latent tuberculosis using TST, among patients suffering from immunomediated inflammatory diseases, was found to be 18.8% [23]. Lower rates of TST positivity in RA may be attributable to the disease itself or the drugs used for its therapy.

Another suggestion from this study was that there is no effect of MTX dose on the results of tuberculin test. However, even the low doses of recent steroid intake significantly reduce the chances of tuberculin positivity. These results echo the results from the past studies. A study carried out at Florence, Italy, revealed that the proportion of positive scoring for TST was significantly lower in patients on treatment with steroids compared with the proportion of positive results in patients who were not receiving treatment with steroids. In the same study, considering the impact of each drug class by multivariate analysis, the use of DMARDs was not found to be associated with test scoring while the use of steroids was associated with a lower probability of a TST or Quantiferon-TB Gold In-Tube (QFT-GIT) positive scoring [23]. North Carolina TB Control Program policy manual states that false negative TST reactions may be caused by high-dose steroids (>15 mg of Prednisone or its equivalent given daily for one month or longer) [24]. Schatz et al. also revealed that in patients treated with daily corticosteroids, tuberculin negativity was associated with a higher dose of corticosteroids [25]. On the contrary, in our study, among patients on steroids within last one month, eight patients were on steroids, equivalent to <15 mg of prednisolone and none of them was found to be tuberculin positive. However, it is not statistically significant due to small number of patients but should be explored further in view of its clinical importance.

In this study, consecutive patients with RA, fulfilling the inclusion criteria, were enrolled from the outpatient department and then grouped according to their therapy. This led to disproportionate groups which is a limitation of the study. Another limitation of the study is that patients were on different forms and doses of different corticosteroids. Long acting immunosuppression may have affected results in some patients. Our data shows that, irrespective of dose or form of steroid taken, percentage of TST positivity is highest in patients not exposed to steroids in last three months (25%), lower in those who had taken steroids in last three months (16%), and lowest in those who were exposed to steroids within one week of TST testing (3%). This intermediate degree of TST positivity in group exposed to steroids in last 3 months but not in last 1 week could be because of differing magnitude and duration of immunosuppressive effect of different forms of steroids taken by the patients.

Despite 2 billion people having latent TB infection, only a fraction (<10 million a year) fall sick with active TB disease [26]. However, in patients with RA, treatment with TNF-*α* inhibitors shows an increased risk of serious life-threatening infections, including reactivation of latent TB infection [27]. Thus screening for active TB and LTBI has become mandatory prior to the initiation of TNF therapies [28, 29] and tuberculin skin test and QFT-GIT remain the largely used tests [30–33]. Bélard et al. showed that prednisolone severely suppressed QFT-GIT and TST performance, whereas the long-acting corticosteroids methotrexate, azathioprine, and 5-ASA did not have a similar detrimental effect and suggested that patients should be screened for LTBI with QFT-GIT or TST prior to initiation of prednisolone therapy and negative QFT-GIT or TST results interpreted with caution in patients treated with any corticosteroid [34]. We endorse this view and are especially concerned for the patients with RA planned for TNF-*α* inhibitor therapy as most of these patients, owing to their high disease activity, are usually already taking steroids in one form or another. In the ATTRACT trial about 60% patients were taking 10 mg or less of corticosteroids at the start of the study [35]. In such a situation TST cannot be relied upon for screening of latent tuberculosis. Our study suggests that steroid intake within the last 1 week significantly lowers the chances of tuberculin positivity. Therefore in patients with RA, before administration of biologicals, tuberculin test should be read with caution if there is history of recent steroid intake.

## Figures and Tables

**Table 1:** Demographic profile of patients grouped according to methotrexate dose.

	Control MTX (*n* = 119)	Group A (*n* = 44)	Group B (*n* = 27)	Group C (*n* = 49)	Group D (*n* = 11)
Age (years) (mean ± SD)	46.1 ± 10.5	46.2 ± 10.9	48.7 ± 11.2	50.7 ± 11.4	43.4 ± 11.1
Sex ratio (M : F)	01 : 07.5	01 : 03.9	01 : 03.5	1 : 06	1 : 10
Smokers, m (%)	24 (20.2)	12 (27.3)	3 (11.1)	15 (30.6)	3 (27.3)
RF positive, m (%)	86 (72.3)	34 (77.3)	14 (51.8)	32 (65.3)	7 (63.6)
Disease duration in months (mean ± SD)	76.4 ± 62.6	60.4 ± 55.0	87.5 ± 66.2	107.5 ± 70.4	74.2 ± 28.8
DAS 28 score (mean ± SD)	5.4 ± 1.4	4.1 ± 1.2	3.9 ± 1.0	4.3 ± 1.2	4.3 ± 1.2

Control MTX: patients who had not taken MTX.

Group A: patients on MTX < 7.5 mg/week.

Group B: patients on MTX > 7.5–15 mg/week.

Group C: patients on MTX > 15–22.5 mg/week.

Group D: patients on MTX > 22.5 mg/week.

**Table 2:** Demographic profile of patients grouped according to steroid intake.

	Control S (*n* = 182)	Group I (*n* = 36)	*P* value
Age (years) (mean ± SD)	48.0 ± 10.5	47.2 ± 10.9	0.66
Sex ratio (M : F)	01 : 06.3	1 : 08	
Smokers, m (%)	39 (21.4)	10 (27.8)	0.41
RF positive, m (%)	126 (69.2)	24 (66.7)	0.76
Disease duration in months (mean ± SD)	85.7 ± 67.1	68.4 ± 52.2	0.089
DAS28 score (mean ± SD)	4.8 ± 1.4	5.1 ± 1.5	0.185
Tuberculin positive, m (%)	45 (24.7)	1 (2.8)	0.002

Control S: patients without any steroid intake within last three months.

Group I: patients with recent steroid intake defined as intake of any dose of steroid in any form (oral, intramuscular, or intra-articular) within 1 week prior to tuberculin test.

**Table 3:** Comparison of tuberculin sensitivity among patients taking different doses of methotrexate (*n* = 250).

Dose specific group	Number of patients with TST positivity (%)	Confidence interval	*P* value
Control MTX (*n* = 119)	23 (19)	10.83, 27.16	0.135
Group A (*n* = 44)	4 (9)	5.26, 32.73	
Group B (*n* = 27)	8 (30)	1.04, 36.95	
Group C (*n* = 49)	14 (29)	6.03, 31.97	
Group D (*n* = 11)	2 (18)	0.00, 50.15	

Control MTX: patients who had not taken MTX.

Group A: patients on MTX < 7.5 mg/week.

Group B: patients on MTX > 7.5–15 mg/week.

Group C: patients on MTX > 15–22.5 mg/week.

Group D: patients on MTX > 22.5 mg/week.

**Table 4:** Comparison of tuberculin sensitivity among RA patients based on steroid exposure (*n* = 250).

Groups based on steroid exposure	Number of patients with TST positivity (%)	Confidence interval	*P* value
Control S (*n* = 182)	45 (25)	17.49, 31.95	0.009
Group I (*n* = 36)	1 (3)	0.00, 9.18	
Group II (*n* = 32)	5 (16)	0.52, 30.72	

Control S: patients without any steroid intake within last three months.

Group I: patients with recent steroid intake defined as intake of any dose of steroid in any form (oral, intramuscular, or intra-articular) within 1 week prior to tuberculin test.

Group II: patients with history of steroid intake within last three months but not in last one week.
